# Predicting global variation in infectious disease severity: A bottom-up approach

**DOI:** 10.1093/emph/eow005

**Published:** 2016-03-14

**Authors:** Per M. Jensen, Henrik H. De Fine Licht

**Affiliations:** Department of Plant and Environmental Sciences, University of Copenhagen, Frederiksberg, Denmark

**Keywords:** case-fatality ratio, mumps, malaria, tuberculosis, leptospirosis, demography

## Abstract

**Background and objectives:** Understanding the underlying causes for the variation in case-fatality-ratios (CFR) is important for assessing the mechanism governing global disparity in the burden of infectious diseases. Variation in CFR is likely to be driven by factors such as population genetics, demography, transmission patterns and general health status. We present data here that support the hypothsis that changes in CFRs for specific diseases may be the result of serial passage through different hosts. For example passage through adults may lead to lower CFR, whereas passage through children may have the opposite effect. Accordingly changes in CFR may occur in parallel with demographic transitions.

**Methodology:** We explored the predictability of CFR using data obtained from the World Health Organization (WHO) disease databases for four human diseases: mumps, malaria, tuberculosis and leptospirosis and assessed these for association with a range of population characteristics, such as crude birth and death rates, median age of the population, mean body mass index, proportion living in urban areas and tuberculosis vaccine coverage. We then tested this predictive model on Danish historical demographic and population data.

**Results:** Birth rates were the best predictor for mumps and malaria CFR. For tuberculosis CFR death rates were the best predictor and for leptospirosis population density was a significant predictor.

**Conclusions and implications:** CFR predictors differed among diseases according to their biology. We suggest that the overall result reflects an interaction between the forces driving demographic change and the virulence of human-to-human transmitted diseases.

## Abstract (precis)

A hundred years ago, infectious diseases disappeared over few decades as major causes of mortality in several Northern European countries. Heart-attack, stroke and cancer then became the main causes of deaths. Why and how this occurred remains unknown, but just possibly it was the natural consequence of demographic change.

## INTRODUCTION

Infectious diseases remain one of the most important causes of morbidity and mortality in many parts of the world [1, 2]. In addition to disease specific and age adjusted mortality and morbidity rates, epidemiological measures of disease ‘severity’ include case-fatality-ratio (CFR= mortality rate/incidence rate) or time-to-death (also equal to survival rate, SR = mortality rate/prevalence rate) [3]. It is the variation in these measures that allows international comparisons of the impact of infectious diseases.

Scholars studying epidemics in the beginning of the 20th century proposed that CFR and SR followed specific patterns where CFRs typically were markedly higher in the early phase of epidemics for a number of diseases [4], and that the epidemics lost momentum as CFR declined. Based on this observation they hypothesized that serial passage was the primary cause of epidemics coming to an end. This view was contested by others who argued that this observation reflected the interaction between susceptible, infected and immune segments of the population [5]. More recently evidence has emerged which would lead us to reconsider the possible importance of the serial passage hypothesis. This includes, CFR variation for measles over the 20th century [6] and the vaccine driven eradication of small pox [7], which both suggest that CFR declined prior to the ‘disappearance’ of a disease. We restated the century old hypothesis i.e. that a substantial proportion of the variation in CFR could be explained by serial passage and its effect on virulence [5].

Here, we hypothesize first that serial passage through children selects for pathogen virulence. Second the probability of achieving continued serial passage through children is likely to decline during demographic transition because of declining birth rates. [8] A reduction in death from infectious diseases would be a natural consequence [9–13].

The aim of the present study was to examine whether country-specific CFRs for a number of infectious human diseases were predictable from vital statistics. This was examined under the assumption that patterns of CFR is mainly driven by bottom up processes i.e. that birth rates for some infectious diseases, can be used to predict disease specific CFR variation.

## METHODS

We explored this hypothesis in a two stage process. Firstly we used contemporary data to explore the influence of population attributes such as age, nutritional status, birth rates and vaccination status. We then tested the predictions from this analysis by an analysis of historical data from Denmark for mumps, malaria and tuberculosis.

### Contemporary data records on CFR and SR

The first step required that records of CFR could be gathered over a wide range of birth rates. The records collected and managed by the WHO, which covers most nations in the world and therefore also populations at widely different stages of demographic development, appeared to be the logical choice as source of information. We explored the data on infectious diseases in the Global Health Observatory of the World Health Organization [14], the Data Presentation System connected to the WHO Mortality Database [15] and also performed our own extracts from the WHO Mortality Database [16] when the data was unavailable from WHO [15, 16] and GHO [14]. Codes (ICD-10) for disease identification and database extractions were retrieved from WHO [17]. Finally information on vaccination coverage was retrieved from WHO [18].

Choosing these WHO databases as source of information carries certain drawbacks. For example, the quality of the extracted data cannot be assumed to be very accurate, rather assessment of mortality statistics by Mathers *et al.* [19] showed that there were only 23 countries with data that were more than 90% complete, i.e. where poorly defined causes of death accounted for <10% of total causes of death. Adding to this, misclassification of causes of death appear frequent in countries such as Sri Lanka and Mexico [20, 21], and can also be quite considerable in developed countries [22]. It should also be accepted that records of morbidity are even more uncertain, because poor access to medical services negatively affects recording of relatively benign infections. Hence the calculated CFRs are associated with considerable uncertainty.

We decided to include only diseases that provided a minimum of 30 records of observations and screened the available databases until we found a set of diseases which included at least two diseases that we believed could be modeled (i.e. their epidemiology changed markedly during the early 20th century epidemiological transition) and two diseases that we did not believe could be modeled (they have less clear or no particular association with the epidemiological transition). As result of this procedure we here evaluate CFR for:

Mumps (caused by the Mumps virus), which like measles is a paramyxovirus that is only directly transmitted through contact between humans [23] due to its very limited survival in the environment (<1 h, [24])*.* It would thus belong to the viruses that before the epidemiological transition were important for human mortality and which later were considered a minor threat to human health. While being preventable by vaccination, mumps out-breaks remain frequent in developed countries [23].

Malaria (caused by protozoan *Plasmodium* sp.) is a vector borne disease transmitted between humans by mosquitos belonging to the genus *Anopheles* [25]. While currently being considered a tropical disease it was also a disease that was widespread in northern Europe in the 18th and 19th century [26–29] where it disappeared during the early 20th century. No vaccination is available and mosquito control remains an important tool to limit the number of malaria cases in endemic regions [25].

Tuberculosis (caused by the bacteria *Mycobacterium tuberculosis)* caused high mortality in Europe during the 17th to 19th century [30]. It fundamentally differs from mumps and malaria however, both by remaining an important disease in most countries and by being carried by a great majority of healthy carriers [31], where only ∼10% will develop active disease as young adults [32]. More than any other disease, tuberculosis has been associated with poverty and a poor standard of living [33, 34]. Also *M. tuberculosis* can survive for more than 100 days in the environment [24], which means that its survival is not directly tied to the survival of the host. It is noteworthy that a number of socio-economic factors are important for tuberculosis risk, e.g. the country of birth will remain a significant risk factor for more than 20 years after immigration to a low risk country [35], which suggests that early life conditions play a role in the epidemiology of tuberculosis.

Leptospirosis (caused by pathogenic bacteria belonging to the genus *Leptospira).* The bacteria have their reservoir in a wide range of mammals and are not transmitted among humans. The infection is mostly mild, but certain types (serovars) e.g. carried by rats are more likely to cause severe diseases [36].

The biology, incompleteness of the records and use of multiple recoding systems raised a number of issues, which had to be addressed prior to statistical analysis. These included: (i) infectious diseases often occur in epidemics, which in given years may occur in one country but not in others. This means that information covering multiple years is required to establish comparable mean incidence rates. (ii) Many countries will in some years not report any cases, while they do so in other years. When calculating the incidence rate it must be assumed that the lack of information is either zero or absent. The choice will affect the calculation when a mean incidence is drawn over several years. (iii) The databases provide case numbers for whole countries but it is not always possible to get information on the size of the population that is exposed. If we simply assume that the entire population is exposed, when it is not, then a biased measure of incidence rate is provided. This obstacle is relevant both when a parasite is only found in parts of the country and when parts of the population are vaccinated. (iv) When using data across recording systems (e.g. both morbidity and mortality) within nations it has to be assumed that they are based on comparable diagnostic criteria, providing equally precise estimates of abundance. If this is indeed true then the pairing of data is likely to be more reliable because ‘often we can greatly increase the precision by making comparisons within matched pairs of experimental material’ [37]. As a result of these considerations, average values were, when possible, drawn over longer periods for all variables to establish mean morbidity and mortality (Table 1). In calculating mortality rates from WHO [16], absent values were accepted as zero values, because it is typically the countries with few cases that in some years are not reported. The mean annual incidence and mortality were calculated as the total number of recorded cases divided by the range of years included. Population numbers for calculation of incidence and mortality rates originated from GHO [14], because WHO [16] had low coverage for recent years.

**Table 1.** Overview of diseases, their classification (ICD-10), occurrence (*N*= number of countries reporting more than zero cases) and morbidity and mortality (rate per 100 000 inhabitants) as means over the given period of years

Disease	ICD-code	Type	N	Mean	SD	CV	Period obtained	Source
Mumps	B26	Morbidity	102	22.24	89.48	4.02	2009–2011	[14]
		Mortality	46	0.01	0.04	4.68	1994–2011	[16]
		CFR^a^	34	0.03	0.14	4.64		
Malaria	B50, B51, B52 B53	Morbidity	102	1494.78	3173.60	2.12	2006–2011	[14]
		Mortality	71	43.08	53.26	1.24	2010	[14]
		CFR^a^	68	0.05	0.14	2.53		
Tuberculosis New and relapse	A16, A17 A18	Morbidity	194	68.00	86.45	1.27	1990–2011	[14]
		Mortality	71	5.34	17.81	3.34	2008–2010	[15]
		CFR^a^	71	0.09	0.07	0.74		
Leptospirosis	A27	Morbidity	51	2.42	6.37	2.64	Recent decades	[54]
		Mortality	71	0.19	0.51	2.71	1999–2009	[16]
		CFR^a^	44	0.09	0.14	1.59		

a CFR values are calculated from natural numbers, while Fig. 1 show the CFR without such adjustment.

### Independent variables

A number of population attributes were retrieved from the GHO [14]: crude birth rate (CBR, births/population size), death rate (DR), median age of the population (MA), mean body mass index (BMI), proportion living in urban areas (PROPU) and from WHO [18], tuberculosis vaccine coverage (VAC) (Table 2). Each of the chosen variables refers to specific attributes of human populations which previously have been hypothesized to be associated with changes in CFR or pathogen virulence. DRs affect virulence because shorter host longevity favors those parasites that leave the host earlier, i.e. are transmitted more quickly [5, 11, 12]. Median age of the population represent the possible effect of risk of mortality varying with age [38–40] and BMI the possible effect of risk of mortality varying with nutritional status [41]. Finally, the proportion of people living in urban areas (PROPU) should allow the observation of density dependence, while the proportion of vaccinated individuals (VAC) should capture the effect of protective immunity and herd effects [5]. Certainly other hypotheses and variables are relevant, but since several of these are not easily considered e.g. the possible interaction between infectious diseases [42], then we accepted that these five alternative variables represented an acceptable statistical challenge to the serial passage hypothesis.

**Table 2.** Summary of variation in independent variable and the variation

Variable	N	Mean	SD	Minimum	Maximum	Period obtained	Source
CBR	183	22.4	10.7	8.4	49.9	2011–2012	[14]
Life expectancy at birth (converted to DR)	194	67.6	9.5	40.8	81.3	1990, 2000 and 2012	[14]
MA	183	28.0	8.5	15.0	45.5	2007–2012	[14]
BMI	179	24.8	2.3	20.1	31.8	2000–2009	[14]
PROPU	194	56.1	23.2	11.0	100.0	2007– 2012	[14]
VAC	165	0.9	0.1	0.2	1.0	1990–2011	[18]

Values represent means over the specified period for the given number of countries (*N*)

CBR was log_e_-transformed to allow for diminishing returns. DR was calculated as 1/life expectancy at birth × 100, which compared to the Crude Death Rate (CDR, deaths/population size) is less influenced by population age structure than actual crude DRs. The calculated DR returned mortality rates that scaled in the same numerical interval as log_e_(CBR) i.e. from 1 to 4, and thus model estimates should be directly comparable. PROPU was here accepted as a measure of population density, which would be comparable across nations without any transformation, and thus the remaining variables were included untransformed.

Cross correlation analysis for examination of potential confounding was performed under PROC CORR (SAS 9.3, SAS Institute) on independent variables and correlation between CFR, morbidity and mortality rates were performed on log_e_-transformed values under PROC CORR. Finally, CFRs were analysed under the PROC GENMOD procedure (distribution = binomial, link= logit) using the DSCALE option to retrieve conservative estimates for *P* values. Here, mean annual morbidity and mortality were rounded to natural numbers to comply with the underlying assumption of the statistical procedure (PROC GENMOD, SAS 9.3, SAS institute) and *P* values <0.01 was accepted as relevant for predictive purposes. We did not include interactions between the six independent variables, because these would have produced out-puts which would have no clear biological meaning e.g. the interaction between urban proportion and vaccination. Neither did we reduce the statistical models because the cross correlation analysis indicated that the independent variables were highly correlated (All 15 tests had *P*’s < 0.001, Table 3). Hence, we only identified significant effects when these were observed in models adjusted for the effects of other relevant variables. Multiple evaluations with various combinations of the independent variables were executed, leading to many different outcomes. While considerable differences were noted among models, it was also noted that differences between diseases remained much the same and the associated conclusions similar. The following presentation included the model with the greatest number of variables, which were deemed more realistic than the models with fewer independent variables. Given that the analysis of global records was less than comprehensive we checked the validity of the main predictors that were identified. This was done by examining the correlation between disease occurrence [43–45] and vital statistics in Denmark [46].

**Table 3.** Cross correlation analysis for the five independent variables (top row: PCC, middle row *P* value, bottom row (*n* = countries)

	DR	MA	BMI	PROPU	VAC
Crude birth rate (CBR)	−0.8918	−0.92003	−0.60859	−0.59194	−0.40843
	<0.0001	<0.0001	<0.0001	<0.0001	<0.0001
	183	183	179	183	157
Life expectancy at birth (DR)		0.82144	0.71401	0.67358	0.43192
		<0.0001	<0.0001	<0.0001	<0.0001
		183	179	194	165
Age median of the population (MA)			0.52	0.61	0.30
			<0.0001	<0.0001	0.0001
			179	183	157
BMI				0.55	0.35
				<0.0001	<0.0001
				179	154
Proportion of the population living in urban area (PROPU)					0.26
					0.0006
					165

## RESULTS

Mumps was observed to have a highly variable CFR ranging from nearly 10^−^^6^ to nearly 1 (Fig. 1a) and the CFR was significantly correlated with both incidence and mortality rate [Pearson’s correlation coefficient (PCC) for *n* = 34: PCC = −0.85, *P* = 0.001 and PCC = 0.69, *P* = 0.001, respectively], suggesting that both measures were contributing to the variability in Mumps CFR. Malaria CFR ranged from ∼10 ^−^^3^ to nearly 1 (Fig. 1b) and the CFR was only correlated with mortality rates (PCC for *n* = 68: PCC = 0.01, *P* = 0.94 and PCC = 0.66, *P* = 0.001, for morbidity and mortality rates, respectively). The range of tuberculosis CFR was quite narrow (Fig. 1c) but had significant correlation with mortality rates (PCC for *n* = 78: PCC = 0.15, *P* = 0.20 and PCC = 0.64, *P* = 0.001, for morbidity and mortality rates, respectively). Lastly, leptospirosis CFR ranged from ∼10 ^−^^5^ to 10 ^−^^2^, which correlated with both morbidity and mortality rates (PCC for *n* = 48: PCC = −0.32, *P* = 0.03 and PCC = 0.59, *P* = 0.001, for morbidity and mortality rates, respectively). Overall, the four diseases appeared to have very different epidemiological patterns.

**Figure 1.**
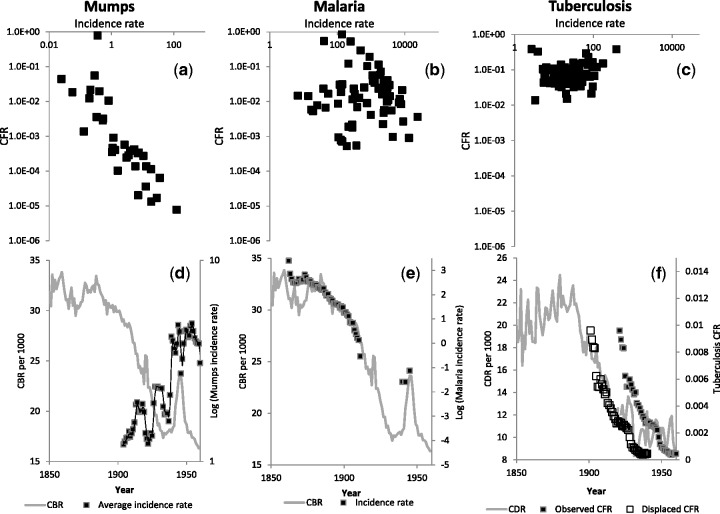
(a) Scatterplot of mumps CFR across global populations versus incidence rates. (b) Scatterplot of malaria CFR across global populations versus incidence rates. (c) Scatterplot of tuberculosis CFR across global populations versus incidence rates. (d) Mumps incidence (averaged over 7 years to remove epidemic waves) and birth rates in Denmark from 1850 to 1950. Mumps records originated from Danmarks Statistik [43]. (e) Incidence of autochthonous malaria and birth rates in Denmark from 1850 to 1950. Malaria records originate from Hansen [44] and Danish Statistical Yearbook [45]. (f) Tuberculosis CFR (number of deaths/number of treated) and DRs in Denmark from 1850 to 1950 both as observed (black squares) and displaced 20 into the past (white squares). Tuberculosis records originated from Danmarks Statistik [43]. CBR and CDR was retrieved from The Human Mortality Database [46]

Birth rate (CBR) and DR were significantly correlated with CFR for several diseases (Table 4), and mumps and malaria was clearly differed from tuberculosis and leptospirosis by having positive effects of CBR and sizable negative intercepts. In contrast, DR was the main variable associated with tuberculosis CFR and only population density, as given by the PROPU areas, was significant for leptospirosis.

**Table 4.** Summary of statistic out-put for the analysis of CFR and SR measures as result of variation in human population characteristics in four infectious diseases

Disease	Parameter	Estimate	Standard error	Wald 95% confidence limits		Wald χ2	Pr > χ2	AIC	N
MumpsCFR	Intercept	−164.208	51.462	−265.071	−63.345	10.18	0.0014	82	34
	Log_e_(CBR)	22.890	8.222	6.774	39.006	7.75	0.0054		
	DR	28.025	19.550	−10.292	66.342	2.05	0.1517		
	PROPU	−0.088	0.054	−0.194	0.019	2.62	0.1057		
	MA	1.033	0.453	0.146	1.920	5.21	0.0225		
	BMI	1.079	0.599	−0.094	2.253	3.25	0.0714		
MalariaCFR	Intercept	−63.220	21.195	−104.762	−21.678	8.9	0.0029	1211524	68
	Log_e_(CBR)	10.302	3.933	2.594	18.011	6.86	0.0088		
	DR	0.629	1.157	−1.638	2.897	0.3	0.5865		
	PROPU	0.015	0.013	−0.010	0.039	1.35	0.2461		
	MA	0.671	0.297	0.089	1.253	5.11	0.0237		
	BMI	0.376	0.222	−0.059	0.812	2.87	0.0904		
TuberculosisCFR	Intercept	−12.739	4.657	−21.867	−3.611	7.48	0.0062	16086	57
	Log_e_(CBR)	0.574	1.017	−1.420	2.568	0.32	0.5728		
	DR	7.945	1.132	5.727	10.163	49.28	<0.0001		
	PROPU	0.002	0.008	−0.013	0.018	0.08	0.7776		
	MA	0.092	0.049	−0.004	0.188	3.52	0.0605		
	BMI	−0.140	0.067	−0.271	−0.010	4.42	0.0356		
	VAC	−1.829	0.998	−3.785	0.126	3.36	0.0668		
LeptospirosisCFR	Intercept	7.664	19.317	−30.196	45.524	0.16	0.6916	767	44
	Log_e_(CBR)	−0.660	2.167	−4.906	3.587	0.09	0.7608		
	DR	4.515	5.643	−6.545	15.574	0.64	0.4237		
	PROPU	0.041	0.010	0.021	0.062	15.83	<0.0001		
	MA	−0.110	0.143	−0.391	0.171	0.59	0.4422		
	BMI	−0.547	0.216	−0.970	−0.123	6.39	0.0115		

## DISCUSSION

The observed correlations might be uncertain because the underlying reporting systems are biased and provide imperfect or flawed indications of the specific epidemiological pattern for the given diseases e.g. underreporting of milder cases. Still it seems unlikely such shortcomings could account for the major differences in CFR ranges, because this requires, not only that a few observations are flawed, but that they all are. Similar arguments are valid for the generalized linear model for CFR, which both due to confounding factors and an absence of relevant variables could be misleading as to the importance of the independent variables. While suspicions of poor accuracy and biases are easily imagined, they are also difficult to prove, and the main method of assessing the validity of the results is therefore to assess the predictive value of the models. Since the main predictor for changes in CFR was clearly identified in all diseases (Table 4) and correlations with incidence rates were characterized, the simplest method of evaluation was to assess these results for consistency with historical records of the disease and its predictor.

For mumps it would appear that CBR predicts variation in CFR (Table 1) and that CFR corresponds to changes in the incidence rate (Fig. 1a). It therefore follows that incidence rates would be negatively correlated with birth rates. This appears to be consistent with Danish crude incidence rates for mumps from 1901 to 1960, which in general increased in the period (Fig. 1d). Mumps thus appears to be a highly predictable disease, which is tightly associated with variation in birth rates. It should be noted that CFR variation as observed in mumps is often accepted as a measure of virulence variation, but in the present case we cannot argue that the variation in CFR only depends on parasite characteristics since age-distributions and associated differences in average immune-competence across populations [38–40], are confounded with differences in CBR. However, even though differences in age structure across populations were a substantial contributor to variation in CFR, it would not necessarily explain all the observed variation (Fig. 1a). The comprehensive review for measles CFR variation given by Wolfson *et al.* [47] indicates no clear variation in CFR with age, while a few studies that evaluated children under the age of 5 years showed a decreasing trend with age. The data for measles indicate a 2- to 3-fold difference among age-groups which—if paralleled in mumps—would mean that perhaps half the observed variation is attributable to differences in age-structure, while the remainder is associated with differences in mumps virus virulence. We would therefore tentatively accept that ‘virulence’ also changed according to birth rate variation [11] and other factors, such as indicated in Table 4.

The correlation between CFR and incidence rates for malaria (Fig. 1b) was less clear than for mumps (Fig. 1a), presumably because malaria is caused by several species and is transmitted by a number of different vectors across the world [25]. The difficulties in documenting a correlation between CFR and incidence rates also arise from the fact that not all people in the given countries are exposed and hence that the incidence measure is severely biased in some countries. Albeit, even a cautious interpretation would lead to the conclusion that mumps and malaria differ fundamentally in their relationship between CFR and incidence rate, and comparing the temporal development in Denmark for mumps and malaria in Denmark from 1901 to 1960 (Fig. 1d and e) would also suggest that the underlying principles are dissimilar—in spite of the similar results in the analysis of CFR (Table 4). The more diverse biology of malaria and inaccurate incidence rate is, however, not the only feasible explanation for the lack of clear correlation between CFR and incidence rate (Fig. 1b), since the lack of clear correlation may be the unavoidable by-product of a limited range in CFR in malaria. Neither do the diverging patterns of mumps and malaria over the first half of the 20th century necessarily mean that the underlying drivers of CFR’s differ (Fig. 1d and e). It should be noted that malaria has high lower-values in the CFR range (Fig. 1b), and that this could be indicative of a lower virulence threshold for disease maintenance, i.e. mumps can through simple contact be transmitted as it does not require any particular dose or level of virulence, while malaria at low inoculation dose in humans [48] or due to other constraints would fail to infect humans. This would be consistent with the variation in global malaria incidence rates, which are lower than 250 cases per 100 000 inhabitants where national birth rates are less than 25 births per 1000 inhabitants, and typically absent where birth rates are <20. A lower threshold would account for the disappearance of malaria in Denmark in 1911–41 and 1945 to the present. Finally, transmission constraints and a threshold in malaria transmission would explain why the two human-to-human transmitted diseases have the same dependency on birth rates in CFR (Table 4), but different resulting occurrence: Mumps has negative correlation, while we suspect that malaria within its limited distribution has a positive correlation (Fig. 1b and d, and ignoring the eight highest CFR values in 1d). A number of authors have also argued that demographic change caused the natural eradication of malaria in European countries under temperatures that could sustain malaria transmission and thus this suggestion has been reached by more conventional epidemiological studies [26–29]. Several of these authors emphasized that demographic change—and not variation in mosquito abundance—were better predictors of the natural eradication of malaria in northern Europe, because it also allowed for the historical occurrence of malaria north of the Arctic Circle, which is not encompassed by conventional ideas on malaria epidemiology.

The epidemiological pattern in tuberculosis differed from mumps and malaria in all aspects that we would expect from the differences in their biology. Interpreting these results requires more caution than for mumps and malaria because for tuberculosis there is a less strict temporal correlation between infection and death. Still, a correlation with CDR is not surprising since historically tuberculosis has been associated with periods of poor living conditions and appeared to recede gradually in Europe in the early part of the 20th century as living conditions improved [33, 34]. Here, we cautiously note that indices of tuberculosis CFR in Denmark (calculated as no. of deaths/number of treated) tracks mortality rate (with a 20 years delay, Fig. 1f), such as it would be inferred from the global analysis and age-related risk [32]. Displacing the data 20 years for this disease, would inspire the idea that not only the place of birth [35] but also the time of birth is a significant contributor to the risk of tuberculosis. Importantly, vaccination was not a major contributor to this early development since systematic vaccination in Denmark began in the 1940’s [49], which would affect the number of recorded cases a few decades later [32].

Because Leptospirosis was mainly characterized by showing relatively high positive association with population density (PROPU), probably due to differences in *Leptospira* reservoirs in urban and rural settings [36], then it appeared that the main predictors for all four diseases fell within particular hypotheses. This conclusion may be difficult to accept since we strongly suspect that the underlying data are unreliable, but we argue that we can place some trust in the results because (i) the analysis incorporate paired-record analysis, that minimize disease specific bias and ‘passively’ remove national records that are not systematically monitoring a given disease, i.e. providing records on both morbidity and mortality and (ii) the effect-size is expanded to such scale (e.g. 10 ^−^^6^ to 1) that even a 5-fold bias in the underlying records, is of little consequence.

## CONCLUSIONS AND IMPLICATIONS

We propose that the idea of serial passage and CFR variation should be reconsidered because demographic changes appeared to accurately predict CFR variation in mumps and malaria in a manner consistent with the hypothesis. Since infectious disease diversity is also strongly correlated with human fertility then it could be argued, that this applies to many diseases [50] which further qualifies the hypothesis. This hypothesis would also in very simple terms explain why Europeans in just a few years will be able to celebrate a centennial without any major epidemics [51] and assist in explaining variable findings on ‘cost of reproduction’ on longevity [52]. The latter because reductions in longevity under this hypothesis would result not only from the number of children born, but also from varying interaction patterns among household members. But more importantly, our results suggest that reductions in birth rates have considerable potential to alleviate disease burdens. Therefore international family planning initiatives, which represent a key instrument to promote global development and health [53], may have an unrecognized collateral benefit.
